# Review of Pharmacological Effects of *Antrodia camphorata* and Its Bioactive Compounds

**DOI:** 10.1093/ecam/nep108

**Published:** 2011-01-03

**Authors:** Madamanchi Geethangili, Yew-Min Tzeng

**Affiliations:** Institute of Biochemical Sciences and Technology, Chaoyang University of Technology, Wufeng, Taiwan

## Abstract

*Antrodia camphorata* is a unique mushroom of Taiwan, which has been used as a traditional medicine for protection of diverse health-related conditions. In an effort to translate this Eastern medicine into Western-accepted therapy, a great deal of work has been carried out on *A. camphorata*. This review discusses the biological activities of the crude extracts and the main bioactive compounds of *A. camphorata*. The list of bioactivities of crude extracts is huge, ranging from anti-cancer to vasorelaxation and others. Over 78 compounds consisting of terpenoids, benzenoids, lignans, benzoquinone derivatives, succinic and maleic derivatives, in addition to polysaccharides have been identified. Many of these compounds were evaluated for biological activity. Many activities of crude extracts and pure compounds of *A. camphorata* against some major diseases of our time, and thus, a current review is of great importance. It is concluded that *A. camphorata* can be considered as an efficient alternative phytotherapeutic agent or a synergizer in the treatment of cancer and other immune-related diseases. However, clinical trails of human on *A. camphorata* extracts are limited and those of pure compounds are absent. The next step is to produce some medicines from *A. camphorata*, however, the production may be hampered by problems related to mass production.

## 1. Introduction

**Figure 1:**
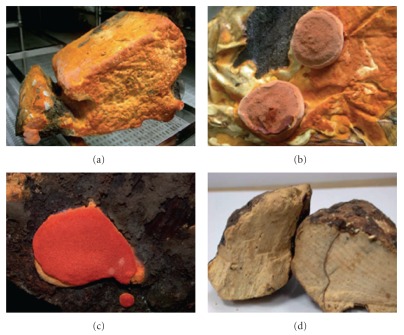
*Antrodia camphorata* from solid-state cultivation of wood. (a) Mycelium from 12-month-old sample. (b) Fruiting bodies from 18-month-old sample. (c) Fruiting bodies from 24-month-old sample. (d) Fruiting bodies from multiple years grown sample.

About 80% of the world population currently relies on indigenous or traditional medicines for their primary health needs, and most of these therapies involve the use of herbal extracts, often in aqueous solutions [1–3]. *Antrodia camphorata* (Syn. *Antrodia cinnamomea*) is a fungal parasite on the inner cavity of the endemic species *Cinnamomum kanehirae* (Bull camphor tree) Hayata (Lauraceae) (Figure 1). The host plant is a large evergreen broad-leaved tree, which only grows in Taiwan, and is distributed over broad-leaved forests at an altitude of 200–2000 m [4]. *Cinnamomum kanehirai* is a high quality material used to manufacture valuable furniture. The government has recently protected this endemic tree species from forest-denudation since this species in nature is relatively rare [5]. In Taiwan, *A. camphorata* is called as “*Niu-chang-chih*” or “*Chang-chih*” or “*Niu-chang-ku*” or “*Chang-ku*” [5]. Locally, it is believed that *A. camphorata* is a present from heaven for Taiwanese and, is a well-known Chinese folk medicine and claimed “ruby in mushroom” in Taiwan [5]. It grows in the mountain ranges of Taoyuan, Miaoli, Nantou, Kaohsiung, Taitung and Hualien of Taiwan [3]. The trophophase of *A. camphorata* occurs from June to October [6]. Being a local species, *A. camphorata* was historically used in Taiwan by the aborigines as a traditional prescription for the discomforts caused by alcohol drinking or exhaustion [5]. Furthermore, the regular consumption is believed to preserve human vitality and promote longevity. The preparations from fruiting bodies have been used for the prevention, or treatment, of numerous diseases including liver diseases, food and drug intoxication, diarrhea, abdominal pain, hypertension, itchy skin and tumorigenic diseases [7, 8]. The aim of this contribution is to review the literature covering pharmacological and phytochemical aspects of *A. camphorata*.

## 2. Taxonomical Description

*Ku* is a Chinese common name meaning mushroom; *chih* means a famous *Ganoderma*-like fungus. *Antrodia camphorata* was first published and identified as new *ganoderma* species, *Ganoderma camphoratum*, by Zang and Su in 1990 [9]. However, according to fruiting-body morphology and cultural characteristics, this fungus has been proposed the name as *A. camphorata* [5, 10]. In 2004, a phylogenetic analysis based on sequence data derived from large ribosomal subunit sequences of ribosomal RNA genes indicated that *A. camphorata* is distantly related to other species in *Antrodia* and, consequently, the fungus was transferred to the new genus *Taiwanofungus* [11]. However, using polymorphism analysis of internal transcribed spacer regions of the ribosomal RNA gene, *A. camphorata* was reconsidered as an *Antrodia* species [12]. The current taxonomic position of *A. camphorata* is as follows [13]: Fungi, Basidiomycota, Homobasidiomycetes, Aphyllophorales, Polyporaceae. Clearly, however, the nomenclature and exact taxonomy (genus and species) of *A. camphorata* is still the subject of debate and needs further research. In this article, we have chosen the name as *A. camphorata* to describe this unique Formosan fungus. The fruiting bodies of *A. camphorata* assume different plate-like, bell-like, hoof-like or tower-like shapes. They are flat on the surface of wood at the beginning of growth. Then the brim of the front edge rises to roll into plate-shaped or stalactites. The top surfaces of *A. camphorata* are lustrous, brown to dark brown in color, with unobvious wrinkles, flat and blunt edges. The bottom sides are orange red or partially yellow with ostioles all over [12]. In addition, *A. camphorata* exhales strong smell of sassafras (camphor aroma), becomes pale yellowish brown when sun-dried and has a strong bitter taste. The red to light cinnamon fruiting bodies of *A. camphorata* are bitter and have a mild camphor scent like the host woods [5]. The mycelia isolated from the fruiting bodies of *A. camphorata* form orange red and orange brown to light cinnamon-colored colonies [5]. The hyphae of *A. camphorata* possess generative hyphae 2–3.5 *μ*m with clamp connections, and hyaline to light brown skeletal hyphae up to 4.5 *μ*m wide with weakly amyloid. Basidia, 12–14 × 3.0–5.0 *μ*m, is clavate and 4-sterigmate with a basal clamp. Basidiospores, 3.5–5.0 × 1.5–2 *μ*m, are cylindrical, hyaline, smooth and sometimes slightly bent [14].

## 3. Ethnomedicine

*Antrodia camphorata* has long been used in traditional medicines of Taiwan for the treatment of twisted tendons and muscle damage, terrified mental state, influenza, cold, headache, fever and many internally affiliated diseases [14]. In 1773, a traditional Chinese medical doctor Wu-Sha found that Taiwan aborigines have often chewed the fruiting bodies and/or decoction of *A. camphorata* for the discomfort caused by excess alcohol or exhaustion because of lifestyle [14]. After that Dr Wu studied the usage of *A. camphorata* based on the locals' experiences, and began to use it to treat diarrhea, abdominal pain, hypertension, itchy skin, viral infection, stomachitis, diabetes mellitus, nephritis, proteinuria, liver cirrhosis, hepatoma, influenza, car sickness, calenture and motion-sickness [7, 15]. After being used for years in Taiwan, the mushroom is now believed to be a potential protecting agent for metabolic syndrome. Recently, many studies have indicated that its medicinal applications go far beyond the original usage. Therefore, demand for the fruiting bodies of *A. camphorata* has far exceeded the supply. Thus, artificial cultivation was developed as a substitute. Currently, *A. camphorata* is available in three ways, gathering in the wild fruiting bodies, wood or solid-state cultivation, and submerged cultivations. Particularly, fruiting bodies and mycelium produced by *A. camphorata* wood or solid-state cultivation instead of gathering in the wilds may solve market's demand. In Taiwan *A. camphorata* is commercially available in the form of fermented wine or pure cultures in powdered, tablet and capsule form [16].

## 4. Chemical Constituents

**Figure 2:**
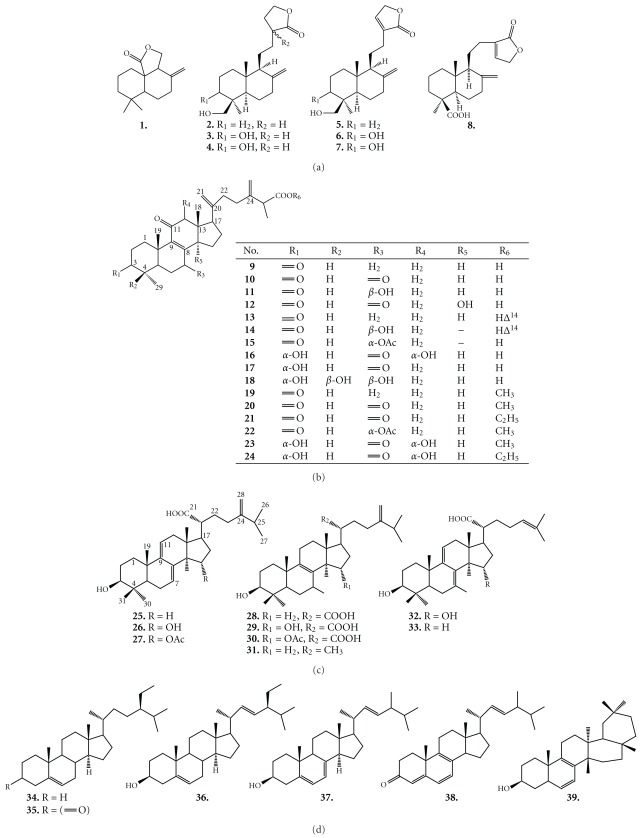

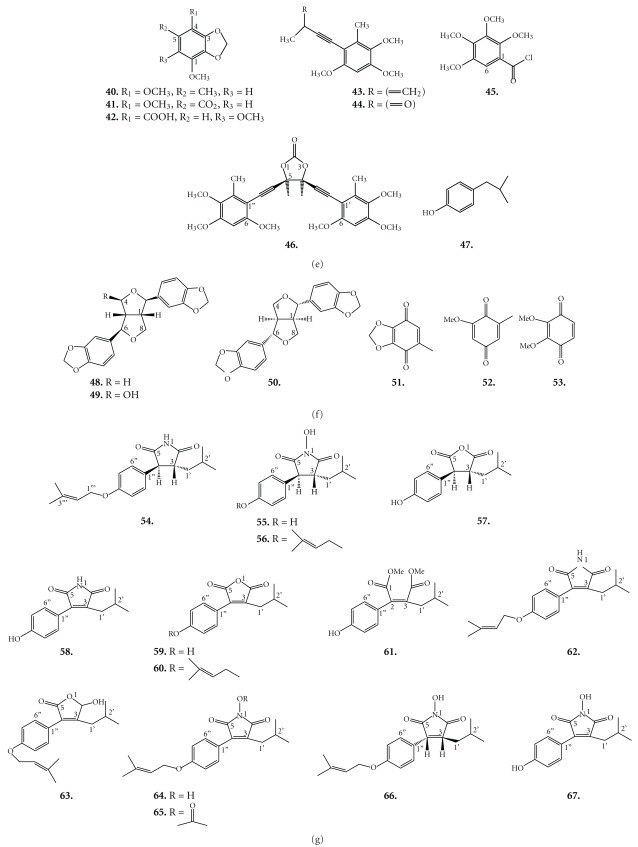

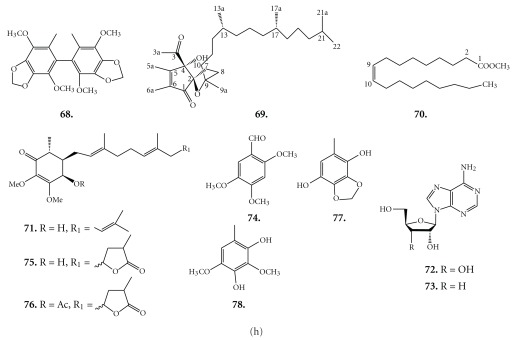
Isolated constituents from A. camphorata. (a) Sesqui- and diterpenoids. (b) Ergostane type triterpenoids. (c) Lanostane-type triterpenoids. (d) Triterpenoid related compounds. (e) Benzenoids. (f) Lignans and benzoquinone derivatives. (g) Succinic and maleic derivatives. (h) Miscellaneous compounds.

A total of 78 compounds have been identified and structurally elucidated. Predominant in fruiting bodies are generally terpenoids in a large number (39 compounds) [17–25], though there are a few publications on the constituents of the solid-state cultivated mycelium and, mycelium from submerged cultivations [26–36]. A large number of triterpenoid compounds (31 structures) with similar or even the same structures were described within the last few years. A common feature of these structures is ergostane or lanostane skeleton. Due to the high amount of 63% of terpenoids in the fruiting bodies of *A. camphorata*, this group of natural compounds has been in the focus of many phytochemical studies. Interestingly, no distinct terpenoid glycosides have ever been isolated from this species; in contrast to polysaccharides that have been elucidated. Furthermore, several other constituents were described from *A. camphorata* comprising benzenoids, lignans, benzoquinones and maleic/succinic acid derivatives, in addition to polysaccharides. Finally, sterols, nucleotides and fatty acids were detected in this species [37–40]. Typical structures and their activities of isolated constituents from *A. camphorata* are depicted in Figure 2 and Table 1, respectively.

## 5. Pharmacological Effects of Crude Extracts

The scientific world's particular interest in *A. camphorata* and its curative properties originated from the realm of traditional medicine. Ethnic medicine has come to be an irreplaceable source of knowledge of medicinal mushrooms and their curative qualities, as well as creating clues for scientific research, which usually confirms the legitimacy of their usage [41, 42]. This part of review will deal with the pharmacological effects of crude extracts of the *A. camphorata* in different models of *in vivo* and *in vitro* studies.

### 5.1. Anti-Cancer Activities

Both the fruiting bodies and mycelium of *A. camphorata* have potent anti-proliferative activity against various cancers *in vitro* and *in vivo*. It was indicated that there were multiple potent mechanisms underlying the anti-cancerous effects of *A. camphorata*. The crude CHCl_3_/MeOH extract from fruiting bodies of *A. camphorata* exhibited significant cytotoxic activity with an IC_50_ value of 4.1 *μ*g ml^−1^ against P-388 murine leukemia cells [18]. The ethylacetate extract from fruiting bodies of *A. camphorata* (EAC) exhibited apoptotic effects in two human liver cancer cell lines, Hep G2 and PLC/PRF/5 in a dose-dependent manner [43]. In addition, EAC also initiated mitochondrial apoptotic pathway through regulation of B-cell lymphoma (Bcl)-2 family proteins expression, release of cytochrome c, and activation of caspase-9 both in Hep G2 and PLC/PRF/5 cells [43]. Furthermore, EAC also inhibited the cell survival signaling by enhancing the amount of I*κ*-*α* in cytoplasm and reducing the level and activity of nuclear factor (NF)-*κ*B in the nucleus, and subsequently attenuated the expression of Bcl-X_L_ in Hep G2 and PLC/PRF/5 cells [43]. Treatment with EAC also caused another human liver cancer cell line Hep 3B to undergo apoptotic cell death by way of calcium-calpain-mitochondria signaling pathway [44]. Another study reported that EAC could inhibit the invasiveness and metastasis of liver cancer cell line PLC/PRF/5 cells through the inhibition of angiogenesis [45]. We previously reported that the CHCl_3_ extract from fruiting bodies of *A. camphorata* (FBAC) showed cytotoxic activity with an IC_50_ value of 22, 150, 65 and 95 *μ*g ml^−1^, against cancer cell lines Jurkat, Hep G2, Colon 205 and MCF 7, respectively. Furthermore, MeOH extract from FBAC also cytotoxic (IC_50_ = 40 *μ*g ml^−1^) to Jurkat cells [46]. *Antrodia camphorata* solid-state cultured mycelium (AC-SS, 1 *μ*g ml^−1^) showed adjuvant anti-proliferative effects with cisplatin (10 *μ*M) or mitomycin (10 *μ*M) in hepatoma cell lines C3A and PLC/PRF/5 cells (*in vitro*) and, on xenografted cells in tumor implanted nude mice (*in vivo*). Furthermore, AC-SS showed its adjuvant effects through the inhibition of MDR gene expressions and the pathway of COX-2-dependent inhibition of AKT phosphorylation [47]. In terms of the very recent literature, Lu et al. [48] noted that ethanol extract from wild fruiting bodies of *A. camphorata* (EEAC) dose-dependently induced human premyelocytic leukemia HL 60 cells apoptosis via histone hypoacetylation, upregulation of histone deacetyltransferase 1 (HDAC 1), and downregulation of histone acetyltransferase activities including GCN 5, CBP and PCAF. Furthermore, combined treatment with 100 nM of trichostatin A (histone deacetylase inhibitor) and 100 *μ*g ml^−1^ EEAC caused synergistic inhibition of cell growth and increase of apoptotic induction through the upregulation of DR5 and NF-*κ*B activation [48].

There are relatively fewer studies on extracts from the solid-state or submerged cultivated mycelium or culture filtrates. Aqueous extract from submerged cultivation mycelium (SCM) of *A. camphorata* exhibited significant cytotoxicity against HL-60 cells but not against cultured human endothelial cells [49]. The SCM resulted dose (25–150 *μ*g ml^−1^) and time-dependent apoptosis, as shown by loss of cell viability, chromatin condensation and internucleosomal DNA fragmentation in HL-60 cells [50]. Furthermore, apoptosis in these cells was accompanied by the release of cytochrome c, activation of caspase-3, specific proteolytic cleavage of poly (ADP-ribose) polymerase (PARP), and also with a reduction in the levels of Bcl-2 [50]. In an another study, the ethanolic extract (0.2–2%, v/v) from solid-state cultivated mycelia of *A. camphorata* showed potent anti-proliferation effect in human non-small cell lung carcinoma A549 cells but not primary human fetal lung fibroblast MRC-5 cells [51]. In addition, this extract triggered the apoptosis in the A549 cells by downregulated human galectin-1, human eukaryotic translation initiation factor 5A, human Rho GDP dissociation inhibitor *α*, human calcium-dependent protease small subunit and human annexin V [51]. To continue, the effects of *A. camphorata* on cancer cells was investigated, methanol extract of SCM exhibited the cytotoxicity in Hep G2 (wild-type p53) and Hep 3B (delete p53) cells with IC_50_ values of 49.5 and 62.7 *μ*g ml^−1^, respectively, after 48 h of incubation. Cell-cycle analysis revealed that the above SCM extract treatment induced apoptosis on Hep G2 via G0/G1 cell-cycle arrest followed by the apoptosis through activation of the caspase-3 and -8 cascades [52]. Furthermore, these authors also reported that the mechanism of MEM-mediated apoptosis in Hep G2 cells through the Fas/Fas ligand (FasL) death receptor pathway [53]. In parallel, Chen et al. [54] noted that the ethanolic extract from SCM has anti-proliferation against Hep G2 and Hep G3 cells with 54.2 and 82.9 *μ*g ml^−1^, respectively. On the other hand, Yang et al. [55] reported that fermented culture broth of *A. camphorata* (FCBAC) exhibits dose (25–150 *μ*g ml^−1^) and time-dependent anti-proliferative effect by induction of apoptosis in breast cancer cell line MCF-7. In addition, this apoptic effect is associated with cytochrome *c* translocation, caspase-3 activation, PARP degradation and dysregulation of Bcl-2 and Bax in MCF-7 cells [55]. These authors also reported that FCBAC has the dose (40–240 *μ*g ml^−1^) and time-dependent apoptotic effect in estrogen-nonresponsive human breast cancer cell line MDA-MB-231 with a similar kind of mechanism as mentioned above [56]. In addition, FCBAC treatment also inhibited the cyclooxygenase (COX)-2 protein expression and prostaglandin E_2_ (PGE_2_) production in MDA-MB-231 cells [56]. Furthermore, FCBAC treatment induced cell-cycle arrest and apoptosis in MDA-MB-231 both *in vitro* and *in vivo* [57]. The *A. camphorata* crude extract (ACCE) at 50 *μ*g ml^−1^ acts as an anti-metastatic agent, by anti-proliferative through induces G2/M cell-cycle arrest followed by suppress the active form of matrix metalloproteinase (MMP)-9 in bladder cancer cell T24 cells [58]. In addition, ACCE (100 *μ*g ml^−1^) showed significant anti-proliferation effect in transitional cell carcinomas (TCC) cell lines RT4, TSGH-8301 and T24 [59]. In RT4 cells, 100 *μ*g ml^−1^ of ACCE showed the p53-independent over expression of p21 followed by downregulation of pRb. On the contrary, treatment with ACCE at 50 *μ*g ml^−1^ resulted in downregulations of Cdc2 and Cyclin B1 in the cell lines TSGH-8301 and T24 [59]. In another study, the ACCE extract at 150 *μ*g ml^−1^ concentration showed anti-cancer effect in androgen responsive prostate cancer cell line LNCaP through pathway Akt→p53→p21→CDK4/cyclin D1→G1/S-phase arrest→apoptosis [60]. In addition, ACCE also inhibited the androgen independent prostate cancer cell line PC-3 through G2/M-phase arrest mediated through pathway p21→cyclin B1/Cdc2 with limited degree of apoptosis [60]. Recently, Lu et al. [61] noted that submerged cultivated *A. camphorata* extract prevents serum-deprived PC-12 cell apoptosis through a PKA-dependent pathway and by suppression of JNK and p38 activities. Ho et al. [62] reported that crude extract of *A. camphorata* (AC) at concentrations of 5–50 *μ*g ml^−1^ did not affect tumor cells PC-3 viability, but at 100–200 *μ*g ml^−1^ decreased viability and induced apoptosis in a concentration-dependent manner. In addition, 25–200 *μ*g ml^−1^ did not alter basal [Ca^2+^]*_i_*, however at 25 *μ*g ml^−1^ decreased the [Ca^2+^]*_i_* induced by ATP, bradykinin, histamine and thapsigargin [62]. The mycelia powder of *A. camphorata* (MAC) at 25–50 *μ*g ml^−1^, did not affect the cell viability in MG63 human osteosarcoma cells, however, at 100–200 *μ*g ml^−1^ decreased viability and induced apoptosis via inhibition of ERK MAPK phosphorylation [63].

In summary, extracts of *A. camphorata* inhibited markedly intracellular signaling and invasive behavior of cancer cells. This complexity can also bring significant advantages. For example, certain components in the natural products can reduce the cytotoxicity of the whole product (and vice versa). Also, the interaction between different biologically active components can be responsible for their effects *in vivo*. Different compounds can modulate unrelated signaling and therefore, can possess synergistic effects [73]. However, the molecular mechanism(s) has not been fully elucidated. Further studies are needed to explore the benefits and safety to cancer patients.

### 5.2. Anti-Inflammatory/Immunomodulatory Effects

Compounds that are capable of interacting with the immune system to up regulate or down regulate specific aspects of the host response can be classified as immunomodulators or biologic response modifiers [74–76]. In peripheral human neutrophils, extracts from SCM of *A. camphorata* displayed anti-inflammatory effects by inhibiting reactive oxygen species (ROS) production with an IC_50_ ranging from 2–20 *μ*g ml^−1^ [77]. The aqueous extract from SCM dose-dependently (25–100 *μ*g ml^−1^) inhibited the lipopolysaccharide (LPS)-induced nitric oxide (NO), tumor necrosis factor (TNF-*α*), interleukin (IL)-1*β* and PGE_2_ production, and inducible nitric oxide synthase (iNOS) and COX-2 protein expression via NF-*κ*B pathway, in macrophages [78]. These results are in parallel to our previous report that CHCl_3_ (3–25 *μ*g ml^−1^) and MeOH (6–50 *μ*g ml^−1^) extracts from fruiting bodies of *A. camphorata* significantly inhibited the enhanced production NO through reducing iNOS expression and, TNF-*α* and IL-12 productions from macrophages [46]. Liu et al. [79] reported that the methanol extract (50 *μ*g ml^−1^) from wild fruiting bodies have more potency than water extracts on the anti-inflammatory activity through inhibiting iNOS, COX-2 and TNF-*α* expression in mouse microglia cell line EOC13.31. In addition, the extracts from solid-state culture were similar to wild-fruiting body in anti-inflammatory activity, but liquid-state fermentation was less effective [79]. To continue, a hot water extracts (fraction MII from mycelium, fractions EII and EIII from culture filtrate) of submerged cultured *A. camphorata* show dose-dependent (5–60 *μ*g ml^−1^) induction of TNF-*α* and IL-6 in peripheral blood culture. Furthermore, these fractions at 20 *μ*g ml^−1^ also showed marked activity in enhancing phagocytosis in human polymorphonuclear neutrophils (PMN), in addition to CD11b upregulation, and monocytes [80]. According to Chang et al. [72] *in vivo* data, hexane extract (100, 200 and 400 mg kg^−1^) from SCM of *A. camphorata* has protection from nephritis by suppression of urine protein and serum blood urea nitrogen levels and decreased the thickness of the kidney glomerular basement membrane in SLE-prone NZB/W F1 mice [72].

### 5.3. Anti-Hepatitis B Virus Replication

It was reported that extracts from the mycelium of *A. camphorata* have *in vivo* anti-hepatitis B virus (HBV) activity in a dose-dependent manner without cytotoxicity [81]. The ethanol extract displayed anti-HBV effects on both wild-type and lamivudine-resistant HBV mutants [71]. The ability of *A. camphorata* to inhibit the replication of HBV *in vivo* and *in vitro* may be one additional reason for considering this fungus as a potential therapeutic for HBV infection.

### 5.4. Anti-Oxidant Activities

Accumulating data have shown that *A. camphorata* is a potent direct free radical scavenger [82–85]. The stable free radical 1,1-diphenyl-2-picrylhydrazyl (DPPH) is scavenged by the extracts of *A. camphorata* [83, 84]. Aqueous extracts of *A. camphorata* inhibited nonenzymatic iron-induced lipid peroxidation in rat brain homogenates with an IC_50_ value of 3.1 mg ml^−1^ [83]. It has been reported that, compared to other *A. camphorata* extracts, the fermented culture broth of *A. camphorata* (FCBA) and aqueous extracts of the mycelia from *A. camphorata* (AEMA) harvested from submerged cultures are the most potent inhibitors of lipid peroxidation, possessing marked free-radical-scavenging activity [82]. The aqueous extract from SCM of *A. camphorata* is possessing anti-oxidant property with respect to oxidative modification of human low-density lipoproteins (LDL) in a time- and concentration-dependent manner [86]. A recent study reported that FCBA and AEMA at 25–100 *μ*g ml^−1^ and 50–200 *μ*g ml^−1^, respectively, possess antioxidant properties in human umbilical vein endothelial cell (EC) culture system [87]. In addition, both FCBA and AEMA treatment significantly inhibited apoptotic cell death in the ECs, as evidenced by reduced DNA fragmentation, cytochrome *c* release, caspase-3 activation and dysregulation of Bcl-2 and Bax [87]. Shu and Lung [85] observed the antioxidant activity (lipid peroxidation, scavenging effect on DPPH radical, hydroxyl free radicals, superoxide anion, reducing power activity and chelating effect on ferrous ions) of methanolic extracts from mycelia and filtrates of *A. camphorata* at two different concentrations (0.2 and 0.6 mg ml^−1^). To continue, 2.5 mg ml^−1^ of methanolic extract irradiated with 20 kGy *γ*-rays showed potent anti-oxidant property by scavenging abilities of 92.3–103% on DPPH radicals [88].

### 5.5. Hepatoprotective Activity

Ao et al. [89] reviewed the potential of *A. camphorata* in treating liver diseases, which provided the major biologically active constituents and their effect or mode of action. The fruiting bodies and mycelium of *A. camphorata* are shown to have protective activity against liver hepatitis and fatty liver induced by acute hepatotoxicity of alcohol [90]. The methanolic extract from wild and solid-state cultures of *A. camphorata* exhibited angiotensin-converting enzyme (ACE) inhibitory activities in spontaneously hypertensive rats [91]. The dry matter of submerged cultivation (DMC) filtrate and aqueous extracts from fruiting bodies have been reported to possess hepatoprotective activity against liver damage induced by CCl_4_ [83, 92]. Both of the extracts reduce glutathione (GSH)-dependent enzymes such as glutathione peroxidase, glutathione reductase and glutathione S-transferase. Histopathological evaluation of the rat liver revealed that the DMC reduced the incidence of liver lesions, including neutrophil infiltration, hydropic swelling and necrosis induced by CCl_4_ [92]. A new formulation comprising the filtrate of *A. camphorata* and extracts from *Astragalus membranaceus*, *Salvia miltiorrhiza* and *Lycimm chinense* found to have significant inhibitory activity against the elevated ALT level in CCl_4_-treated animals to an extent that was even better than when the filtrate was used alone [93].

In conclusion, the reported data showed that *A. camphorata* may exert its hepatoprotective effects, though different mechanisms such as scavenging free radicals responsible for cell damage, enhancing the enzymes responsible for antioxidant activity, inhibiting the inflammatory mediators and/or induction of the regeneration of the liver cells. Previously reported data revealed that *A. camphorata* is a potent free radical scavenger [82–85]. It is therefore possible that hepatoprotective action of *A. camphorata* is partially due to its antioxidant activity. The antioxidant activities of the filtrate and mycelium extracts were correlated with the presence of total polyphenols, crude triterpenoids and the protein/polysaccharide ratio of the crude polysaccharides [82]. Aqueous extracts of *A. camphorata* inhibited non-enzymatic iron-induced lipid peroxidation in rat brain homogenates with an IC_50_ value of about 3.1 mg ml^−1^ [83]. These results suggest that *A. camphorata* exerts effective protection against chemical-induced hepatic injury *in vivo* by free radical scavenging activities.

### 5.6. Prevention of Liver Fibrosis

Using CCl_4_-treated rats as an experimental model, it is found that the filtrate of fermented mycelium from *A. camphorata* has the preventive and curative properties of liver fibrosis [94]. Post treatment with mycelium to CCl_4_-administered rats clearly accelerated the reversal of fibrosis and lowered the elevated mRNA levels of hepatic collagen I, transforming growth factor (TGF)-*β*1 and tissue inhibitors of matrix metalloproteinase (TIMP)-1. Furthermore, it is confirmed that hepatic lipid peroxidation is increased during fibrogenesis where hepatic malondialdehyde (MDA) and hydroxyproline (HP) contents in curative groups were remarkably restored [94]. Another study reported that fermented mycelium is effective in reversing liver fibrosis induced by dimethylnitrosamine (DMN), while the lowered activities of antioxidative enzymes (SOD, catalase and GSH-Px) in the liver were not restored [95]. Therefore, more *in vivo* studies and randomized controlled clinical studies should be performed to further elucidate the mechanisms of action of *A. camphorata*.

### 5.7. Neuroprotective Effect

It is reported that mycelium extract from submerged cultivation of *A. camphorata* prevents serum-deprived PC-12 cell apoptosis through PKA-dependent pathway and by suppression of JNK and p38 activities [61, 96].

### 5.8. Antihypertensive Effect

The extracts of wild and solid-state cultures *A. camphorata* were obtained by sequential extraction with cold water (CWS), methanol (MS) and hot water (HWS), respectively. Among these three, only extract MS (10 mg kg^−1^ BW) showed potent antihypertensive effects in spontaneously hypertensive rats by decreased systolic blood pressure and diastolic blood pressure, however these effects were absent in Wistar Kyoto rats [97]. These results might have a scope to develop *A. camphorata* to be a healthy (or functional) food to regulate blood pressure.

### 5.9. Vasorelaxation Effect

The SCM extract of strain B85 shown to have concentration-dependent vasorelaxation with maximal relaxation of 40.34 ± 7.53% through an endothelium-dependent mechanism, whereas strains 35 398, 35 396 and B71 had mild effects in isolated rat aortic rings [98]. In conclusion, preclinical and clinical studies are necessary for the validation of this natural product in the prevention and/or therapy of above mentioned applications. Also, the effects of isolated compounds require to be tested further as discussed subsequently.

## 6. Bioactivities of Isolated Compounds

### 6.1. Terpenoids

The bitter components of *A. camphorata* are triterpenoids and have known pharmacological activities (Table 1). Triterpenes are considered to be potential anti-cancer agents due to activity against growing tumors, they have direct cytotoxicity against tumor cells rather than to normal cells. Cultivated mycelium has been reported to contain similar compounds with wild fruiting bodies [8]. Biological study revealed that zhankuic acids A (**10**) and C (**16**) exhibited cytotoxic activity against P-388 murine leukemia cells with an IC_50_ value of 1.8 and 5.4 *μ*g ml^−1^, respectively [18]. However, the molecular mechanism(s) responsible for the inhibitory effects have not been fully elucidated. We reported that the isolates (**10**, **16**, **20**, **25**–**27**, **29** and **32**) from fruiting bodies of *A. camphorata* showed inhibitory effects on *Spodoptera frugiperda* S*f*9 insect cells where zhankuic acids A (**10**) and C (**16**) and methyl antcinate B (**20**) being most potent [64]. In continuation of our studies on the activities of pure compounds from fruiting bodies of *A. camphorata*, these eight compounds together with antcins A (**9**) and C (**11**) were examined for their cytotoxic data against various cancer cell types. The three zhankuic acids, **10**, **16** and **20** displayed the tumor-specific cytotoxicity with an IC_50_ range from 22.3 to 75.0 *μ*M against the colon, breast, liver and lung cancer cell lines [65]. One of the most potent triterpene was methyl antcinate B (**20**). Furthermore, compounds **10**, **16** and **20** demonstrated to induce apoptosis in HT-29 cells, as confirmed by sub-G1 cell-cycle arrest as well as DNA fragmentation. Furthermore, the expression of poly-(ADP-ribose) polymerase cleavage, Bcl-2 and procaspase-3 were also suppressed, in addition to their synergistic cytotoxic effect (4 *μ*M each) in HT-29 cells [65]. Our previous results state that the chloroform extract of *A. camphorata* demonstrated inhibitory activity on colon cancer cells (see previously). Analysis suggested that the active principles *in vivo* were triterpenoids. These results indicate that the triterpenoids fraction of *A. camphorata* may be a useful ingredient in the treatment of colon cancer. To continue, our results also reveal that compounds **9**, **18** and **20** displayed potential anti-*Helicobacter pylori* activity and its associated inflammation in human gastric epithelial AGS cells, by inhibition of adhesion and invasion, NF-*κ*B activation and the subsequent release of IL-8 in AGS cells [99].

Antcins A (**9**), B (**10**) and eburicol (**31**) have anti-inflammatory activity by inhibition of *N*-formylmethionyl-leucyl-phenylalanine (fMLP)-induced superoxide generation in human neutrophils with an IC_50_ value of 8.5, 9.8 and 50.5 *μ*M, respectively [30]. To continue, antcin C (**11**), dehydroeburicoic acid (**25**) and eburicoic acid (**28**) also noted for their immuno-modulating activity by reduced ROS in the above mentioned system with IC_50_ values of 16.9, 144.8 and 43.9, respectively [67]. The compounds **10**, **17** and **16** and, antcin K (**18**) isolated from ethanol extracts of wild fruiting body has shown concentration-dependent (1–25 *μ*M) anti-inflammatory effects (by modulation of leukocyte activity and inhibition of ROS) induced by fMLP and TPA in human neutrophils [66]. The diterpenoid compounds **2**, **3**, **4**, **5** and **6** isolated from the fruiting bodies of *A. camphorata* have neuroprotective activity in cortical neurons from the cerebral cortex of Harlan Sprague-Dawley rat pups by 39.2, 35.0, 36.7, 30.6 and 27.0%, respectively, at concentrations between 5 and 20 *μ*M [28].

### 6.2. Maleic and Succinic Acid Derivatives

Nakamura et al. [24] noted the cytotoxic data of five new maleic and succinic acid derivatives from the mycelium of *A. camphorata* in LLC tumor cells. The compounds antrodins A (**60**) and D (**56**) had no activity whereas antrodins B (**62**) and C (**64**) had cytotoxic activity with ED_50_ values of 7.5 and 3.6 *μ*g ml^−1^, respectively [24]. Furthermore, the compounds antrodins A–E (**56**, **60**, **62**, **64** and **66**) from mycelium noted to have anti-hepatitis activity [27]. The succinic derivative **54** isolated from fruiting bodies exert both immunostimulatory and anti-inflammatory effects by increased spontaneous TNF-*α* secretion from unstimulated RAW264.7 cells, in addition to suppressed IL-6 production (IC_50_ = 10 *μ*g ml^−1^) in LPS-stimulated cells. Furthermore, the compounds, **57**, **58** and **61** suppressed IL-6 production in LPS-stimulated cells with IC_50_ values of 17, 18 and 25 *μ*g ml^−1^, respectively [31]. Antrocinnamomins A (**65**) isolated from mycelium of *A. camphorata* noted to have inhibition of NO production of macrophages [33]. To continue, the tested maleic and succinic-acid derivatives **60**, **62**, **64, 56** and **66** (antrodins A–E) showed HCV protease inhibitory activity with IC_50_ values of 0.9, >100, 2.9, 20.0 and 20.1 *μ*g ml^−1^, respectively [98].

### 6.3. Polysaccharides

Polysaccharides represent a structurally diverse class of biological macromolecules with a wide-range of physicochemical properties. Polysaccharides of *A. camphorata* have been reported to be composed of a variety of monosaccharides, galactose, glucose, mannose, glucosamine and galactosamine [38]. The majority of anti-tumor **β**-d-glucans isolated from *A. camphorata* are **β**-(1→3)-d-glucopyranans and characteristic **β**-(1→6)-d-glucosyl branches [38].

Scientific investigations concerning the inhibition of anti-HBV activity by polysaccharides from fruiting bodies and cultured mycelia of *A. camphorata* were reported in 2002 [38]. Polysaccharides from strain B86 at a dosage 50 *μ*g ml^−1^ exhibited the highest anti-hepatitis B surface antigen effect, which was higher than that of *α*-interferon at a concentration of 1000 U/ml [38]. It is interesting to note that the anti-HBV activity has not been reported for polysaccharides from any other mushroom. Thus, further studies on the relationship between specific polysaccharide fraction and their biological activities are required. Recently, extensive studies on the immunomodulatory and anti-tumor effects polysaccharides from different sources have been reported [100]. For example, a partially purified polysaccharide inhibited the proliferation of human leukemic U937 cells via activation of human mononuclear cells [101]. In addition, these *in vitro* anti-tumor activity was substantiated by the *in vivo* study in sarcoma 180-bearing mice where the intraperitoneal and oral administration of 100 and 200 mg kg^−1^ significantly suppressed the tumor growth with the inhibition rate of 69.1% and 58.8%, respectively [101]. Polysaccharides isolated from *A. cinnamomea* reported to have anti-angiogenic activities in endothelial cells, by dose-dependent inhibition of cyclin D1 expression through vascular endothelial growth factor receptor signal pathway [102]. To continue, Han et al. [103] reported that a neutral polysaccharide named *ACN2a* from the hot water extract of the mycelium of *A. camphorata* to have *in vivo* hepatoprotective activity in mouse model of liver injury that was induced by *Propionibacterium acnes*-LPS [103]. Another study reported that the polysaccharide fractions (from SCM), AC-1, AC-2, AC-3, AC-4 and AC-5 belonged to the category of glycoprotein with mean molecular mass in the range of 394–940 kDa showed anti-inflammatory activity in macrophages [40]. At a concentration of 1 *μ*m, polysaccharides AC-1 and AC-2 showed DPPH radical scavenging activity by 74.5 and 50.5%, respectively. In addition, AC-2 dose-dependently (50–200 *μ*g ml^−1^) inhibit the LPS-induced NO production and iNOS protein expression in macrophages [40]. Polysaccharides from SCM possess immunomodulatory activity by modulating the pro-inflammatory cytokines [104], through inducing Th1-type cytokines such as IFN-*γ* and TNF-*α* in a time-dependent manner but not of Th2 cytokines [70]. A recent study reported that 3–6 weeks oral administration with 2.5 mg of polysaccharides derived from *A. camphorata* (AC-PS) modulate the expression of Th1 cytokines in splenocytes as well as the type1 differentiation of T and B lymphocytes, in addition to reduce the infection rate of *Schistosoma mansoni* in mice [105]. Furthermore, water-soluble polysaccharides (200 *μ*g ml^−1^) from the fermented filtrate and mycelia of *A. cinnamomea* significantly reduced the oxidative DNA damage and ROS induced by hydrogen peroxide in Chang liver cells [106]. A recent study for the first time reported the sulfated polysaccharides (SPSs) from submerged cultivation medium of *A. cinnamomea*. These SPSs dose-dependently inhibited *in vitro* Matrigel tube formation in an angiogenesis model, in addition to their prevention of serum-deprived apoptosis in neuronal-like PC-12 cells [107].

### 6.4. Compounds with Miscellaneous Biological Activities

Among the 10 pure compounds (in concentration range of 5–50 *μ*M) which included one biphenyl, four ergostane- and five lanostane derivatives tested for anti-viral activity against wild-type (HBsAg) and lamivudine-mutant (HBeAg) HBV, the only one biphenyl compound (**68**) at a 50 *μ*M suppressed HBsAg and HBeAg levels by 54.2 and 32.2%, respectively [71]. The compound adenosine isolated from ethanolic extract of SCM, noted it acts through adenosine A_2A_ receptors to prevent rat PC-12 cells from serum deprivation-induced apoptosis [37]. A benzenoid 2,4,5-trimethoxybenzaldehyde (**74**) produced by submerged cultivation of *A. camphorata* reported to has COX-2 inhibitory activity [31]. The compounds antrocamphin A (**43**) and 2,3-dimethoxy-5-methyl[1, 4]benzoquinone (**53**) inhibit the fMLP-induced superoxide generation in human neutrophils with an IC_50_ value of 9.3 and 26.1 *μ*M, respectively [30]. Antroquinonol (**71**), a ubiquinone derivative isolated from mycelia and the fruiting bodies of *A. camphorata* reported to has cytotoxic activities against cancer cell lines MCF-7, MDA-MB-231, Hep 3B, Hep G2 and DU-145, LNCaP with the IC_50_ values ranged from 0.13 to 6.09 *μ*M [32]. In addition, **71** at 256 *μ*M significantly inhibited the production of TNF-*α* and IL-1*β* by 75 and 78%, respectively, in RAW 264.7 cells [72]. Furthermore, compound **71** also noted as potent inhibitor in the synthesis of HBsAg and HBeAg [108]. The compounds antroquinonol B (**75**), 4-acetyl-antroquinonol B (**76**), 2,3-(methylenedioxy)-6-methylbenzene-1,4-diol (**77**) and 2,4-dimethoxy-6-methylbenzene-1,3-diol (**78**) and antrodin D (**56**) from mycelium of *A. camphorata* inhibit NO production in LPS-activated macrophages with an IC_50_ values of 16.2, 14.7, *∼*18, 32.2 and 26.3 *μ*g ml^−1^, respectively [36]. A benzenoid compound **40** has dose-dependent (50–150 *μ*M) anti-proliferation activity in human colon cancer cell line COLO 205 through G0/G1 cell-cycle arrest and induction of apoptosis (>150 *μ*M). In addition, cell-cycle arrest is associated with a significant increase in levels of p53, p21/Cip1 and p27/Kip1, and a decrease in cyclins D1, D3 and A [68].

## 7. Summary and Outlook

This review summarized important areas of investigation being performed on *A. camphorata* with particular emphasis on crude extracts and isolated compounds. Some correlation between the ethnomedical employment and the pharmacological activities has been duly observed in the present review. *Antrodia camphorata* extracts from its fruiting bodies, mycelium and cultivation filtrate showed multiple cancer preventive and anti-inflammatory activities. In addition, these extracts provide a variety of anti-cancer and anti-inflammatory active secondary metabolites and polysaccharides. Of particular promise, due to their potent cytotoxic activity against a number of cancer cell lines, are the triterpenoids with ketonic functional groups. In fact, these triterpenoids, which have also been found in a small number of other mushrooms, are currently under active investigation as potential therapeutic leads [109]. Because the antioxidant action is also a means of lowering chronic anti-inflammatory action, *A. camphorata* hold potential in functional food approaches aimed at normalizing metabolic syndrome.

In the search for active compounds from *A. camphorata*, the majority of research has been performed on extracts from the fruiting bodies and mycelium and, there have been fewer studies on extracts from the submerged cultivated medium. Further studies would be desirable to isolate useful new secondary metabolites by varying cultivation conditions. The pharmacological studies so far have mostly been performed *in vitro* and *in vivo* with animals. Therefore, clinical studies are needed in order to confirm traditional wisdom in the light of a rational phytotherapy. Nevertheless, the former reports could be considered as providing leads for more scientific research. The biological activities of the pure compound administrated or consumed alone were found to be lower than those obtained from the original mixture of active ingredients present in natural medicines including *A. camphorata*. Thus, the combined, synergistic effects of a mixture of active components that are present in *A. camphorata* on biological activities need to be thoroughly assessed. Finally, though we recently developed a cyclodextrin-modified capillary electrophoresis method for the separation and analysis of achiral and chiral triterpenoids from fruiting bodies of *A. camphorata* [110], there however, is a need to establish suitable quality parameters and analytical methods to determine active compounds.

## Figures and Tables

**Table 1:** Chemical constituents and their reported activities of *A. camphorata*.

No.	Compound name	Source	Biological activity	Ref.
	*Terpenoids*			
**1**	Antrocin	F		[19]
**2**	19-Hydroxylabda-8(17)-en-16,15-olide	F	*In vitro* neuroprotective	[28]
**3**	3*β*,19-Dihydroxylabda-8(17),11E-dien-16,15-olide	F	*In vitro* neuroprotective	[28]
**4**	13-*epi*-3*β*,19-Dihydroxylabda-8(17),11E-dien-16,15-olide	F	*In vitro* neuroprotective	[28]
**5**	19-Hydroxylabda-8(17),13-dien-16,15-olide	F	*In vitro* neuroprotective	[28]
**6**	14-Deoxy-11,12- didehydroandrographolide	F	*In vitro* neuroprotective	[28]
**7**	14-Deoxyandrographolide	F		[28]
**8**	Pinusolidic acid	F		[28]
**9**	Antcin A	F	*In vitro* anti-inflammatory, anti-insecticidal and cytotoxic	[30, 64, 65]
**10**	Antcin B (Zhankuic acid A)	F	*In vitro* anti-inflammatory, anti-insecticidal and cytotoxic	[18, 30, 64–66]
**11**	Antcin C	F	*In vitro* anti-inflammatory and cytotoxic	[65, 67]
**12**	Antcin D (Zhankuic acid F)	F		[23]
**13**	Antcin E	F		[21]
**14**	Antcin F	F		[21]
**15**	Antcin G	F		[21]
**16**	Antcin H (Zhankuic acid C)	F	*In vitro* anti-inflammatory, anti-insecticidal and cytotoxic	[18, 64–66]
**17**	Antcin I (Zhankuic acid B)	F	*In vitro* anti-inflammatory	[66]
**18**	Antcin K	F	*In vitro* anti-inflammatory	[66]
**19**	Methyl antcinate A	F		[25]
**20**	Methyl antcinate B	F	*In vitro* anti-insecticidal and cytotoxic	[64, 65]
**21**	Zhankuic acid D	F		[25]
**22**	Methyl antcinate G	F		[21]
**23**	Methyl antcinate H	F		[21]
**24**	Zhankuic acid E	F		
**25**	Dehydroeburicoic acid	F	*In vitro* anti-inflammatory, anti-insecticidal	[64, 65, 67]
**26**	Dehydrosulphurenic acid	F	*In vitro* anti-insecticidal and cytotoxic	[64, 65]
**27**	15*α*-Acetyl-dehydrosulphurenic acid	F	*In vitro* anti-insecticidal and cytotoxic	[64, 65]
**28**	Eburicoic acid	F	*In vitro* anti-insecticidal and cytotoxic	[64, 65]
**29**	Sulphurenic acid	F	*In vitro* anti-insecticidal and cytotoxic	[64, 65]
**30**	Versisponic acid D	F		
**31**	Eburicol (24-methylenedihydrolanosterol)	F	*In vitro* anti-inflammatory	[30]
**32**	3*β*, 15*α*-Dihydroxy lanosta-7,9(11),24-triene-21-oic acid	F	*In vitro* anti-insecticidal and cytotoxic	[64, 65]
**33**	3*β*-Hydroxy lanosta-	F		[17]
**34**	*β*-Sitosterol	F		[28]
**35**	*β*-Sitostenone	F		[28]
**36**	Stigmasterol	F		[28]
**37**	Ergosterol	F		[28]
**38**	Ergosta-4,6,8(14)22-tetraen-3-on3	F		[30]
**39**	*epi*-Friedelinol	F		[30]
	*Benzenoids*			
**40**	1,4-Dimethoxy-2,3-methylenedioxy-5-methylbenzene	F	*In vitro* cytotoxic	[68]
**41**	1,4-Dimethoxy-2,3-methylenedioxy-5-benzoate	F		[24]
**42**	1,6-Dimethoxy-2,3-methylenedioxy-4-benzoic acid	F		[24]
**43**	Antrocamphin A	F	*In vitro* anti-inflammatory	[30]
**44**	Antrocamphin B	F		[30]
**45**	2,3,4,5-Tetramethoxybenzoyl chloride	F		[30]
**46**	Antrodioxolanone	F		[30]
**47**	Isobutylphenol			[34]
	*Lignans*			
**48**	(+) Sesamin	F		[20]
**49**	4-Hydroxy sesamin	F		[20]
**50**	(−) Sesamin	F		[20]
	*Benzoquinone derivatives*			
**51**	5-Methyl-benzo(1,3)-dioxole-4,7-dione	M		[20]
**52**	2-Methoxy-5-methyl(1,4)benzoquinone	M	*In vitro* anti-oxidant	[20]
**53**	2,3-Dimethoxy-5-methyl(1,4)benzoquinone	M	*In vitro* anti-inflammatory	[20, 30]
	*Succinic and Maleic derivatives*			
**54**	*trans*-3-Isobutyl-4-[4-(3-methyl-2-butenyloxy)phenyl]pyrrolidine-2,5-dione	F	*In vitro* anti-inflammatory	[33]
**55**	*trans*-1-Hydroxy-3-(4-hydoxyphenyl)-4-isobutylpyrrolidine-2,5-dione	F	*In vitro* anti-inflammatory	[33]
**56**	3R*,4S*-1-Hydroxy-3-isobutyl-4-[4-(3-methyl-2-butenyloxy)phenyl]pyrrolidine-2,5-dione (antrodin D or Camphorataimide E)	F, M	*In vitro* anti-inflammatory, anti-HBV and anti-HCV	[29, 33, 69]
**57**	*cis*-3-(4-Hydroxyphenyl)-4-isobutyldihydrofuran-2,5-dione	F	*In vitro* anti-inflammatory	[33]
**58**	3-(4-Hydroxyphenyl)-4-isobutyl-1*H*-pyrrole-2,5-dione	F	*In vitro* anti-inflammatory	[33]
**59**	3-(4-Hydroxyphenyl)-4-isobutylfuran-2,5-dione (Antrocinnamomin C)	F	*In vitro* anti-inflammatory	[33, 35]
**60**	3-Isobutyl-4-[4-(3-methyl-2-butenyloxy)phenyl]furan-2,5-dione (antrodin A or Camphorataanhydride A)	M	*In vitro* anti-HBV and anti-HCV	[29, 69]
**61**	Dimethyl 2-(4-hydroxyphenyl)-3-isobutylmaleate	F	*In vitro* anti-inflammatory	[33]
**62**	3-Isobutyl-4-[4-(3-methyl-2-butenyloxy)phenyl]-1*H*-pyrrole-2,5-dione (Antrodin B or Camphorataimide B)	M, B	*In vitro* anti-inflammatory, anti-HBV and anti-HCV	[26, 29, 69]
**63**	Antrocinnamomin D	M		[35]
**64**	3-Isobutyl-4-[4-(3-methyl-2-nyloxy)phenyl]-1*H*-pyrrol-1-ol-2,5-dione (antrodin C or) Camphorataimide C)	M	*In vitro* anti-inflammatory, anti-HBV and anti-HCV	[28, 31, 70]
**65**	Antrocinnamomins A	M	*In vitro* anti-inflammatory	[37]
**66**	3R*,4R*-1-Hydroxy-3-isobutyl-4-[4-(3-methyl-2-butenyloxy)phenyl]pyrrolidine-2,5-dione (Antrodin E or Camphorataimide D)	M	*In vitro* anti-HBV and anti-HCV	[31, 70]
**67**	Antrocinnamomins B	M	*In vitro* anti-inflammatory	[37]
	*Miscellaneous compounds*			
**68**	2,2′,5,5′-Tetramethoxy-3,4,3′,4′-bi-methylenedioxy-6,6′-dimethylbiphenyl	F	*In vitro* anti-HBV	[71]
**69**	*α*-Tocospiro B	F		[30]
**70**	Methyl oleate	F		[20]
**71**	Antroquinonol	M, F	*In vitro* cytotoxic, anti-inflammatory, anti-HBV	[12, 32, 72]
**72**	Adenosine	M	Prevention of PC 12 cells apoptosis	[37]
**73**	Cordycepin	M		[37]
**74**	2,4,5-trimethoxybenzaldehyde	M	Prevention of PC 12 cells apoptosis	[31]
**75**	Antroquinonol B	M	*In vitro* anti-inflammatory	[36]
**76**	4-acetyl-antroquinonol B	M	*In vitro* anti-inflammatory	[36]
**77**	2,3-(methylenedioxy)-6-methylbenzene-1,4-diol	M	*In vitro* anti-inflammatory	[36]
**78**	2,4-dimethoxy-6-methylbenzene-1,3-diol	M	*In vitro* anti-inflammatory	[36]

F: Fruiting bodies; M: Mycelium; B: Culture broth.
